# What predicts the clinical benefits of PARP inhibitors in platinum-sensitive recurrent ovarian cancer: A real-world single-center retrospective cohort study from China

**DOI:** 10.3389/fonc.2022.955124

**Published:** 2022-08-18

**Authors:** Depu Zhang, Shuo Li, Xinxin Zhang, Jingwei Peng, Shiqian Zhang

**Affiliations:** ^1^ Department of Obstetrics and Gynecology, Qilu Hospital of Shandong University, Jinan, China; ^2^ Department of Gynecology Oncology, Shandong Cancer Hospital and Institute, Shandong First Medical University and Shandong Academy of Medical Sciences, Jinan, China; ^3^ Department of Radiation Oncology and Shandong Provincial Key Laboratory of Radiation Oncology, Shandong Cancer Hospital and Institute, Shandong First Medical University and Shandong Academy of Medical Sciences, Jinan, China

**Keywords:** olaparib, niraparib, PARP inhibitor, ovarian cancer, prognosis, real world

## Abstract

**Objective:**

This study assessed the real-world application, effectiveness, and safety of olaparib and niraparib as maintenance therapies in patients with platinum-sensitive recurrent ovarian cancer (PSROC) in China and investigated clinical factors associated with prolonged benefits of poly ADP-ribose polymerase (PARP) inhibitors to help guide clinician treatment-decision making in daily practice.

**Methods:**

This real-world single-center retrospective cohort study was conducted at the Shandong Cancer Hospital and Institute. Archival data of consecutive patients diagnosed with PSROC who achieved a complete response (CR) or partial response (PR) after the last platinum-based chemotherapy and treated with olaparib or niraparib as maintenance therapy from August 2018 to September 2021 were collected.

**Results:**

Overall, 106 women were included in the cohort. Seventy-two (68%) patients were treated with olaparib, while 34 (32%) received niraparib; 99.1% of the patients were diagnosed with high-grade serous carcinoma, and 73.6% had FIGO stages III–IV. Approximately 71.7% of the patients had received PARP inhibitors after the second platinum-based line and 44.3% of the patients achieved a CR in their last platinum-based therapy. The median platinum-free interval (PFI) after the penultimate platinum-based therapy was 10 (95% CI: 10–13.6) months. The median PFS was 21 (95% CI: 13–24.5) months and the median CFI was 22 (95% CI: 16–26.5) months. Consistent with the univariate analysis, the multivariate analysis identified three independent factors associated with prolonged progression-free survival (PFS) and chemotherapy-free interval (CFI): breast cancer susceptibility gene (BRCA) mutant type (p = 0.005 and p = 0.003); PFI ≥12 months (p = 0.01 and p = 0.006); and CR to last platinum-based therapy (p = 0.016 and p = 0.019). It was found that there was no appreciable difference in any grade 3–4 hematological AE between patients who received olaparib and niraparib.

**Conclusion:**

Maintenance treatment with olaparib and niraparib is effective and well tolerated for PSROC patients in real-world clinical practice. Three clinical factors were identified that predicted prolonged survival under maintenance therapy with PARP inhibitors: BRCA mutant type, PFI ≥12 months, and CR to last platinum-based therapy. These findings should be further confirmed with an appropriately powered analysis in studies with larger sample sizes.

## Introduction

Ovarian cancer is a leading cause of cancer-related deaths in women, with an estimated 313,959 new cases and 207,252 deaths each year worldwide (1). At present, the incidence of ovarian cancer in China is on the rise, ranking third among gynecological malignancies with the highest mortality rate (2). Although primarily sensitive to platinum-based chemotherapy, most patients experience recurrence and disease progression within 2 years of treatment (3).

Recently, evidence from several randomized controlled trials (RCTs), including Study19 (4), SOLO2 (5), NOVA (6), and NORA (7), revealed that the incorporation of poly ADP-ribose polymerase (PARP) inhibitors as maintenance therapy for patients with platinum-sensitive recurrent ovarian cancer (PSROC) after the response to the last platinum-based therapy is effective in extending progression-free survival (PFS) and chemotherapy-free interval (CFI).

However, results from randomized controlled trials (RCTs) are often difficult to replicate in a real-world setting (8). In RCTs, investigators reduce bias by using randomization and strict inclusion and exclusion criteria, which may rule out a particular group of patients commonly seen in clinical practice. As a result, the populations enrolled in RCTs can differ significantly from those found in daily practice. Consequently, retrospective analyses are essential to define the clinical benefit of new therapies in broader, everyday cancer populations in different settings, countries, or healthcare systems.

Several retrospective studies have investigated the effectiveness and safety data of olaparib in patients with PSROC in the real-world setting (9, 10). A common limitation of these studies is that only patients with the BRCA mutant type receiving olaparib treatment were included. However, more than 70% of Chinese patients with ovarian cancer did not have a BRCA mutation (11), and a large proportion of patients with PSROC received PARP inhibitor maintenance therapy without BRCA gene testing.

In one cohort, 13.3% of the patients received olaparib after stable disease (SD), 8.8% had PSROC with a non-serous subtype, and 9.7% were platinum-resistant [9]. Together, these findings are very inconsistent with clinical practice. Maintenance therapy with PARP inhibitors is not recommended in platinum-resistant patients or in cases with stable disease according to the Response Evaluation Criteria in Solid Tumors (RECIST 1.1). Patients diagnosed with different histological types of ovarian cancer other than high-grade serous or endometrioid should not receive PARP inhibitors. Nonetheless, the prognostic factors identified based on the above cohorts may mislead clinicians into making inappropriate clinical decisions.

In this study, the effectiveness and safety of olaparib and niraparib were investigated in a real-world setting using a real-life cohort in line with general clinical practice to identify factors associated with long-term benefits of treatment with PARP inhibitors to better guide clinicians in everyday practice.

## Materials and methods

### Patient population

This real-world single-center retrospective study was conducted at the Shandong Cancer Hospital and Institute. This study was approved by the ethics committee.

Study participants included women diagnosed with invasive epithelial ovarian, fallopian tube, or primary peritoneal carcinoma (collectively referred to as ovarian cancer), who achieved a complete response (CR) or partial response (PR) to the last platinum-based chemotherapy and were treated with PARP inhibitors (olaparib or niraparib) as maintenance therapy. Patients diagnosed with borderline epithelial tumors or mucinous carcinoma or who had received PARP inhibitors within a clinical trial were excluded. Archival data from consecutive patients from August 2018 to September 2021 were collected.

The study was conducted in accordance with the principles of the Declaration of Helsinki (12) and the guidelines of the International Conference on Harmonization of Good Clinical Practice. Due to the retrospective design and anonymized data collection of the study, the requirement for informed consent of patients was waived.

### Response criteria and outcome measures

The primary objective of our study was to assess the clinical factors associated with the benefits of PARP inhibitors in a real-world setting. The effectiveness of the treatment was measured using the PFS and CFI. PFS was defined as the time in months from the initiation of PARP inhibitors to the date of progression of the disease according to RECIST 1.1 or the last follow-up. CFI was defined as the time from the end of the most recent platinum-based treatment to the beginning of the next anticancer treatment (excluding maintenance therapy). The overall response to chemotherapy was defined according to RECIST 1.1 and the Gynecological Cancer Intergroup (GCIG) criteria for CA-125. The platinum-free interval (PFI) was defined as the time between the completion of the penultimate platinum-based chemotherapy cycle and the date of the next relapse or progression. The CA-125 response was defined as at least a 50% reduction in CA-125 levels from a pretreatment sample according to the GCIG criteria. Patients were evaluated according to CA-125 only if they had a pretreatment sample that was at least twice the upper limit of the reference range.

The safety of PARP inhibitor treatment was evaluated by adverse events (AEs), which were classified according to the Common Terminology Criteria for Adverse Events (CTCAE) version 5.0.

### Data collection

Clinical data, including patient demographics, clinicopathological characteristics, residual disease after primary surgery, PFI, secondary cytoreductive surgery, recurrence status, and so on, were collected from medical records. BRCA mutation status was reported on case report forms after local testing. A predicted harmful, or suspected deleterious, BRCA mutation based on either blood or tumor testing was defined as BRCA mutant type. No BRCA mutations or BRCA variants of unknown significance were defined as the BRCA wild type. Missing information was supplemented by telephone follow-up or face-to-face inquiries (only for patients who were alive and accessible). The incidence of AEs, as well as dose reductions, dose interruption, and discontinuation of treatment due to AEs were recorded.

### Treatment and follow-up

Olaparib was administered orally at a starting dose of 300 mg twice daily in tablet form and continued until disease progression if toxicities were manageable. Based on the results of the retrospective RADAR analysis (13) of the NOVA study and the fact that the weight of almost all patients with ovarian cancer in China was less than 77 kg, all patients initiated maintenance treatment with niraparib at a fixed starting dose of 200 mg once a day in daily practice at our hospital. Modification of the dosage of olaparib and niraparib was performed at the discretion of the clinicians.

Patients were advised to visit every month for prescriptions, symptom evaluation, and laboratory tests (complete blood count and CA-125 at a minimum) and every 3 months for tumor evaluation (imaging studies, mostly computed tomography scan) until objective disease progression or intolerable toxicities.

### Statistical methods

No formal sample size calculation was performed since the study was exploratory. Continuous variables were reported as means and ranges. Categorical data were reported as frequency and percentage and compared with the chi-square test or Fisher’s test, as appropriate. Survival analyses were conducted using the Kaplan–Meier method and log-rank test. For multivariate analyses, Cox proportional hazard regression analyses were conducted, and hazard ratios (HRs) and 95% confidence intervals (CIs) were calculated. Candidate clinical factors with statistical significance in the univariate analysis were then included in the multivariate analysis. Furthermore, prognostic factors of interest identified in previous studies, such as PARP inhibitors and CA-125 response, were also included. Independent prognostic variables for PFS and CFI were identified using a backward selection procedure. Missing data were excluded from the univariate analyses and were assigned as unknown for the multivariate analysis.

Statistical analyses were performed using IBM SPSS statistics software (version 26.0; IBM Corp., Armonk, NY, USA, RRID : SCR_016479) and Prism software (GraphPad, La Jolla, CA, USA, RRID : SCR_002798). A P-value of <0.05 was considered statistically significant.

## Results

### Patients characteristics

**Figure 1:**
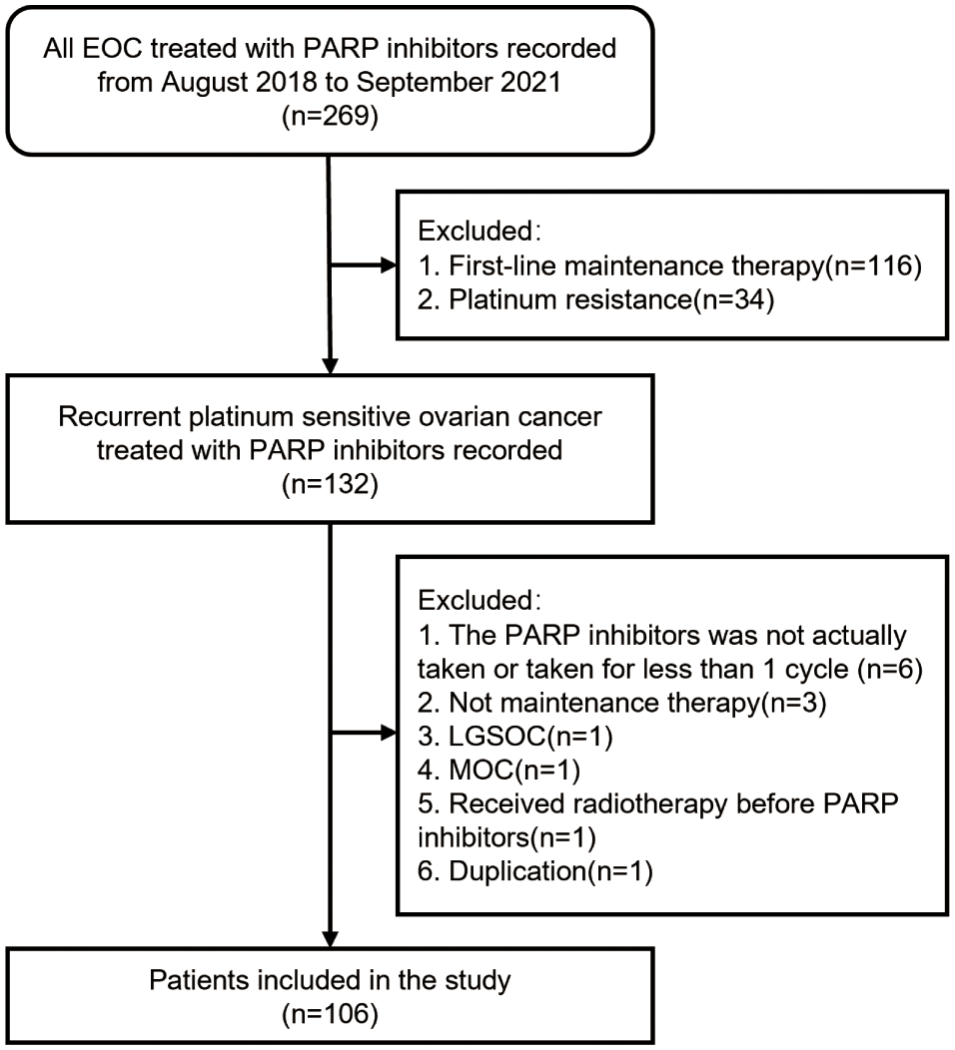
Enrollment flow diagram. Abbreviations: PARP, Poly ADP-ribose Polymerase; LGSOC, low-grade serous ovarian cancer; MOC, mucinous ovarian cancer.

Between August 2018 and September 2021, the clinical files of 269 patients treated with olaparib or niraparib at the Shandong Cancer Hospital and Institute were retrieved for study eligibility, and finally 106 women were included in the cohort according to the inclusion and exclusion criteria (**Figure 1**). Seventy-two (68%) patients were treated with olaparib, whereas 34 (32%) received niraparib.

Patient demographics and clinical characteristics at diagnosis are summarized in **Table 1**. The median age at the time of diagnosis was 54 (a range of 30–73) years. Approximately 99.1% of the patients were diagnosed with high-grade serous carcinoma, 73.6% had FIGO stage III–IV, and 63.2% had no macroscopic residual disease after debulking surgery. About 70% of the patients had KPS scores of 90, whereas 30% of the patients had a KPS score of 80. Comorbidities were reported in 24.5% of the patients. Only 7.6% of the patients had a personal history of breast cancer, and 13.2% of the patients had a familiar history of ovarian or breast cancer.

**Table 1:** Characteristics of patients at diagnosis of ovarian cancer.

	Entire cohort N = 106	Olaparib N = 72	Niraparib N = 34	p
**Age**	54 (30–73)	54 (30–72)	52.5 (39–73)	0.88
<55	55 (51.9)	37 (51.4)	18 (52.9)	0.88
≥55	51 (48.1)	35 (48.6)	16 (47.1)	
**BRCA mutation status**				
Mutant type	23 (21.7)	19 (26.4)	4 (11.8)	**<0.01**
Wild type	42 (39.6)	21 (29.2)	21 (61.7)	
Unknown	41 (38.7)	32 (44.4)	9 (26.5)	
**KPS**				
80	32 (30.2)	19 (26.4)	13 (38.2)	0.21
90	74 (69.8)	53 (73.6)	21 (61.8)	
**Comorbidities**				
Yes	26 (24.5)	17 (23.6)	9 (26.5)	0.75
No	80 (75.5)	55 (76.4)	25 (73.5)	
**Personal history of Breast cancer**				
Yes	8 (7.6)	5 (6.9)	3 (8.8)	0.71
No	98 (92.4)	67 (93.1)	31 (91.2)	
**Familiar history for Breast or Ovarian cancer**				
Yes	14 (13.2)	12 (16.7)	2 (5.9)	0.22
No	92 (86.8)	60 (83.3)	32 (94.1)	
**Familiar history of other cancers**				
Yes	27 (25.5)	18 (25.0)	9 (26.5)	0.87
No	79 (74.5)	54 (75.0)	25 (73.5)	
**Neoadjuvant chemotherapy**				
Yes	40 (37.7)	27 (37.5)	13 (38.2)	0.94
No	66 (62.3)	45 (62.5)	21 (61.8)	
**Histology**				
HGSOC	105 (99.1)	71 (98.6)	34 (100.0)	>0.99
Endometrioid carcinoma	1 (0.9)	1 (1.4)	0 (0.0)	
**FIGO staging**				
I	12 (11.3)	9 (12.5)	3 (8.8)	0.80
II	12 (11.3)	9 (12.5)	3 (8.8)	
III	62 (58.5)	42 (58.3)	20 (58.8)	
IV	16 (15.1)	9 (12.5)	7 (20.6)	
Unknown	4 (3.8)	3 (4.2)	1 (2.9)	
**Macroscopic residual disease**				
Absent	67 (63.2)	44 (61.1)	23 (67.7)	0.80
Present	35 (33.0)	25 (34.7)	10 (29.4)	
Unknown	4 (3.8)	3 (4.2)	1 (2.9)	

KPS, Karnofsky Performance Status; HGSOC, high-grade serous ovarian cancer.

Bold values denote statistical significance at the P < 0.05 level.

Patient characteristics before the administration of PARP inhibitors are summarized in **Table 2**. Overall, 71.7% of patients received PARP inhibitors after the second platinum-based line and 25.5% of patients combined with bevacizumab in the last platinum-based therapy. Approximately 17.9% of patients underwent secondary cytoreductive surgery. A CR was achieved in 44.3% of the patients, and 55.7% had a PR for their last platinum-based therapy according to RECIST 1.1 and the GCIG criteria. A CA-125 response according to the GCIG criteria was observed in 40.6% of the patients. The median PFI after the penultimate platinum-based therapy was 10 (95% CI: 10–13.6) months. Patients with PFI of more than 12 months and 6–12 months were equally distributed.

**Table 2:** Characteristics of patients at time of PARP inhibitors administration.

	Entire cohort N = 106	Olaparib N = 72	Niraparib N = 34	p
**Number of previous lines of platinum-based therapy**				
2	76 (71.7)	49 (68.1)	27 (79.4)	0.23
≥3	30 (28.3)	23 (31.9)	7 (20.6)	
**Secondary cytoreductive surgery**				
Yes	19(17.9)	12(16.9)	7(20.6)	0.79
No	86(82.1)	59(83.1)	27(79.4)	
**Combined with bevacizumab in the last platinum-based therapy**				
Yes	27 (25.5)	15 (20.8)	12 (35.3)	0.11
No	79 (74.5)	57 (79.2)	22 (64.7)	
**Overall response to the last platinum-based therapy**				
PR	59 (55.7)	43 (59.7)	16 (47.1)	0.22
CR	47 (44.3)	29 (40.3)	18 (52.9)	
**CA-125 response**				
Yes	43 (40.6)	25 (34.7)	18 (52.9)	0.12
No	54 (50.9)	39 (54.2)	15 (44.2)	
Unknown	9 (8.5)	8 (11.1)	1 (2.9)	
**PFI after the penultimate platinum-based therapy**				
6–12 months	53 (50.0)	38 (52.8)	15 (44.1)	0.41
≥12 months	53 (50.0)	34 (47.2)	19 (55.8)	

PFI, platinum-free interval.

There were no differences between the olaparib and niraparib groups regarding age (p = 0.88), histological subtype (p >0.99), residual disease (p = 0.80), FIGO stage (p = 0.80), number of previous lines of platinum-based therapy (p = 0.23), PFI (p = 0.41), CA-125 response (p = 0.12), or the overall response (p = 0.22). The only statistically significant difference was the proportion of patients with BRCA wild type (29.2% *vs* 61.7%, p <0.01).

### Survival

With a median follow-up of 17.5 (95% CI: 13-22) months, 49 patients had received PARP inhibitors for at least 12 months at the time of analysis. The median PFS since the start of PARP inhibitor treatment was 21 (95% CI: 13–24.5) months and the median CFI was 22 (95% CI: 16–26.5) months.

**Figure 2:**
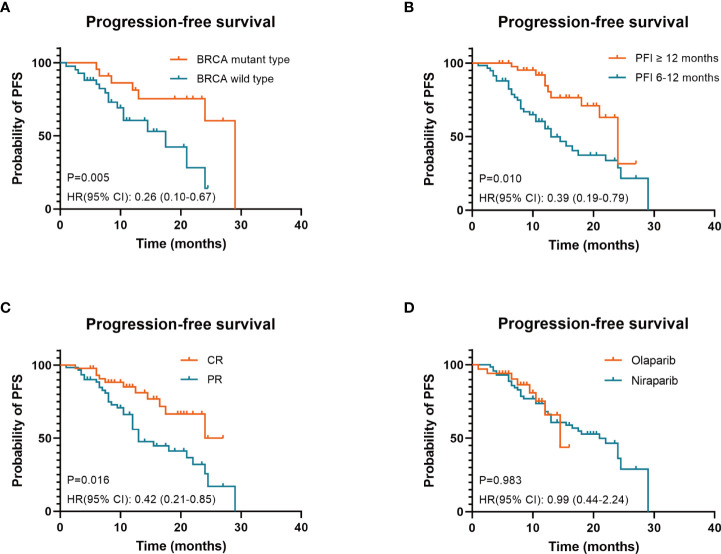
Three factors associated with prolonged progression-free survival (PFS) under PARP inhibitors in patients with platinum-sensitive recurrent ovarian cancer were identified: BRCA mutant type **(A)**, PFI ≥12 months **(B)** and CR to last platinum-based therapy **(C)**. PARP inhibitor regimen was not an independent prognostic factor for PFS **(D)** BRCA, breast cancer susceptibility gene; PARP, poly ADP-ribose polymerase; PFI, platinum-free interval; CR, complete response; PR, partial response; HR hazard ratios; CI, confidence intervals.

Survival analysis was performed (see **Table 3** and **Figure 2** for PFS; **Table S1** and **Figure S1** in the Supplementary Appendix for CFI). Consistent with previous studies, there was no impact by PARP inhibitor regimen (**Figure 2D** and **Figure S1D**), age, FIGO stage, macroscopic residual disease in primary surgery, secondary cytoreductive surgery, bevacizumab administration during the last platinum-based therapy in either PFS or CFI. Interestingly, contrary to previous studies (9, 10), no differences in PFS or CFI were recorded according to the previous platinum-based therapy line and CA-125 response according to the GCIG criteria.

**Table 3:** Univariate and multivariate analysis of progression-free survival for the entire cohort.

Clinical factors	Univariate analysis		Multivariate analysis	
	*HR (95% CI)*	*P*	*HR (95% CI)*	*P*
**PARP inhibitors**				
Olaparib *vs* Niraparib	1.08 (0.47–2.17)	0.983		
**Age**				
<55 *vs* ≥55	0.87 (0.47–1.58)	0.625		
**BRCA mutation**				
Mutant *vs* Wild	**0.37 (0.17–0.83)**	**0.014**	**0.26 (0.10–0.67)**	**0.005**
Unknown *vs* Wild	0.79 (0.41–1.53)	0.471		
**Stage**				
III–IV *vs* I–II	0.65 (0.31–1.33)	0.175		
**Macroscopic residual disease**				
Absent *vs* Present	0.84 (0.43–1.64)	0.591		
**Number of previous lines of platinum-based therapy**				
2 *vs* ≥3	1.18 (0.63–2.20)	0.590		
**Secondary cytoreductive surgery**				
Yes *vs* No	0.88 (0.38–2.00)	0.761		
**PFI after the penultimate platinum-based therapy**				
≥12 months *vs* 6–12 months	**0.35 (0.19–0.64)**	**0.002**	**0.39 (0.19–0.79)**	**0.010**
**Overall response to last platinum-based therapy**				
CR *vs* PR	**0.41 (0.23–0.75)**	**0.007**	**0.42 (0.21–0.85)**	**0.016**
**CA-125 response**				
Yes *vs* No	0.67 (0.36–1.25)	0.186		
**Combined with bevacizumab in last platinum-based therapy**				
Yes *vs* No	1.18 (0.58–2.40)	0.643		

PARP, Poly ADP-ribose Polymerase; PFI, platinum-free interval.

Bold values denote statistical significance at the P < 0.05 level.

In the univariate analysis, factors associated with prolonged PFS and CFI were BRCA mutant type (p = 0.014 and p = 0.008), PFI ≥12 months (p = 0.002 and p = 0.001), and CR to the last platinum-based therapy (p = 0.007 and p = 0.007).

Consistent with the univariate analysis findings, the multivariate analysis identified three independent factors associated with prolonged PFS and CFI: BRCA mutant type (p = 0.005 and p = 0.003), PFI ≥12 months (p = 0.01 and p = 0.006), and CR to the last platinum-based therapy (p = 0.016 and p = 0.019).

Explorative subgroup analysis was performed in the olaparib group (see **Table S2** and **Figures S2A–C** for PFS; **Table S3** and **Figures S2D–F** in the Supplementary Appendix for CFI). These results were consistent with those of the entire cohort. In the olaparib subgroup analysis, factors associated with prolonged PFS and CFI were BRCA mutant type (p = 0.021 and p = 0.018), PFI ≥12 months (p = 0.058 and p = 0.039) and CR to the last platinum-based therapy (p=0.032 and p=0.036). Because of the small sample size, subgroup analysis was not performed in the niraparib group.

### Toxicity

Eighty-six of 106 patients (81.8%) experienced a hematological AE of any grade (see **Table 4**). Hematological AEs of any grade that occurred in either group included thrombocytopenia (26.5% in the niraparib group *vs*. 9.7% in the olaparib group, p = 0.02), leucopenia (73.5% *vs*. 48.6%, p = 0.02), anemia (67.7% *vs*. 68.1%, p = 0.97) and neutropenia (50.0% *vs*. 38.9%, p = 0.28).

**Table 4:** Hematologic AEs and dose adjustment.

	Entire cohort N = 106 (%)	Olaparib N = 72 (%)	Niraparib N = 34 (%)	p
**Hematologic AEs of any grade**				
**Total**	86 (81.8)	58 (80.6)	28 (82.4)	0.83
Leucopenia	60 (56.6)	35 (48.6)	25 (73.5)	**0.02**
Neutropenia	45 (42.5)	28 (38.9)	17 (50.0)	0.28
Anemia	72 (67.9)	49 (68.1)	23 (67.7)	0.97
Thrombocytopenia	16 (15.1)	7 (9.7)	9 (26.5)	**0.02**
**Hematologic AEs of grades 3–4**				
**Total**	19 (17.9)	16 (22.2)	3 (8.8)	0.11
Leucopenia	8 (7.6)	5 (6.9)	3 (8.8)	0.71
Neutropenia	4 (3.8)	3 (4.2)	1 (2.9)	>0.99
Anemia	13 (12.3)	11 (15.3)	2 (5.9)	0.22
Thrombocytopenia	3 (2.8)	2 (2.8)	1 (2.9)	>0.99
**Dose reduction**	19 (17.9)	11 (15.3)	8 (23.5)	0.30
**Dose interruption**	23 (21.7)	15 (20.8)	8 (23.5)	0.75
**Dose discontinuation**	3 (2.8)	3 (4.2)	0 (0)	0.55

AEs, adverse events. Bold values denote statistical significance at the P < 0.05 level.

No grade 3 or worse-severity hematological AE was observed in more than 10% of the patients, except for anemia (in 15.3% of the patients receiving olaparib). It was found that there was no appreciable difference in any grade 3–4 hematological AE between patients receiving olaparib and niraparib.

Dose interruptions were reported in 23 (21.7%) patients. A dose reduction was also implemented in 19 (17.9%) of the patients. Three patients taking olaparib were discontinued because of hematological TEAE (1 for anemia, 2 for thrombocytopenia and anemia), and no discontinuation was required for patients taking niraparib due to hematological AE.

## Discussion

Previous clinical trials have focused more on the survival benefits obtained from PARP inhibitors than with placebos and have paid little attention to the clinical factors associated with prolonged survival. This retrospective study was the first to use a Chinese cohort to assess the prognostic factors of PARP inhibitors as maintenance therapy in patients with PSROC in a real-world setting. We identified three independent clinical factors associated with prolonged PFS and CFI of PARP inhibitors in patients with PSROC: BRCA mutant type, PFI ≥12 months, and CR to the last platinum-based therapy.

Undoubtedly, the BRCA mutation status is an independent risk factor for prognosis that can be fully demonstrated by the activity of PARP inhibitors or by the large amount of clinical trial data. The median PFS was significantly longer in patients with the BRCA mutant type than in patients with the wild type, as reported in previous randomized trials [Study 19 (4), NOVA (6), and NORA (7)].

Consistent with previous studies (9, 10, 14), this analysis indicates that CR to last platinum-based therapy and PFI ≥12 months, which are predictors of improvement in platinum sensitivity, are independent factors for survival benefits with PARP inhibitors. The clinical significance of CR and PFI has already been well established for women with PSROC (9, 14, 15).

Although the GCIG CA-125 response and progression criteria have become increasingly popular in clinical trials of ovarian cancer for more than a decade (16, 17), the prognostic role of CA-125 for maintenance therapy with PARP inhibitors in ovarian cancer is controversial (18, 19). Analyses based on cohorts published by other groups, in which CA-125 levels in more than 30% of the patients were not within the reference range, suggest that normalization of CA-125 is an easy tool for clinicians to predict the duration of treatment with PARP inhibitors (9, 10). In fact, the proportion of cases without normalization of CA-125 in patients who achieved CR or PR from the ultimate platinum-based chemotherapy is much less than 30% in routine practice (20). In our patient cohort derived from real-life clinical practice, the vast majority (94.3%) of our cohort had a CA-125 reading within the normal range after response to platinum-based chemotherapy prior to maintenance therapy.

According to the GCIG criteria (18), we introduced the CA-125 response as a factor in the univariate and multivariate analysis to replace the normalization of CA-125, and data from our study showed that the relative benefit of the PARP inhibitor is evident independently of the CA-125 response. It should be stressed again that currently there are no data to validate the implications of achieving the CA-125 response with respect to PFS in maintenance therapy (18).

Although cross-comparisons should be made with caution, the median PFS of maintenance therapy after platinum-sensitive recurrence was significantly lower than that of maintenance therapy after first-line chemotherapy (21, 22). Conversely, patients who maintained a response to platinum-based treatment after multiple-lines of chemotherapy were thought to be more sensitive to platinum, which was associated with more clinical benefit from PARP inhibitors (23). One study from Italy (10) reported that in patients treated after three or four lines of chemotherapy, patients had a significantly shorter PFS (HR: 2.5, 95% CI 1.3–4.8, p = 0.004). Conversely, another study from France (9) reported that there was no difference in the PFS according to the different number of previous chemotherapy lines (HR: 1.0, 95% CI 0.6–1.8, p = 0.98). Our data confirmed that the number of previous lines did not influence the survival benefits of PARP inhibitors, as demonstrated in the SOLO2 trial (5).

As the first drug targeted therapy for ovarian cancer, olaparib was approved as a maintenance treatment for PSROC patients by the Chinese Food and Drug Administration in September 2018 (24). Niraparib was subsequently approved in December 2019. Within the healthcare coverage system, olaparib and niraparib are the most widely used PARP inhibitors in daily practice in China. Influenced by the different study designs of the NOVA (6) (all comer) and SOLO2 (5) (BRCA mutation selected populations) trials, patients with BRCA wild type tended to choose niraparib more often.

In the olaparib-treated patients, the median PFS was comparable to that of the SOLO2 study (5) (21 months *vs* 19.1 months). Interestingly, all patients enrolled in the SOLO2 study had a BRCA mutation, but in this study, the proportion of patients with a definite BRCA mutation was only 26.4%. Another retrospective study from China achieved similar results in which only 35% of the enrolled cases had a BRCA mutation, but the 12-month PFS rate was similar (63.8% *vs* 65%) (25). Based on the above results, further research and exploration are needed to determine whether Chinese populations can benefit more from olaparib than other races.

The median PFS of the niraparib group in our study was shorter than that reported in the NORA study conducted in the Chinese population (14.5 months *vs* 18.3 months) (7). This can be explained by a lower proportion of BRCA mutant types (11.8% *vs* 36.7%). A higher proportion of patients with more than two lines of prior chemotherapy (20.6% *vs* 0%) also contributed to this difference.

The incidence of non-hematological toxicities was not reported in our study because it is difficult for physicians to record all symptoms in a real-world setting unless informed by the patient, which could result in an underestimation of the real incidence.

The percentage of grade 3–4 hematological AEs, dose reduction (15.3%) and treatment discontinuation (4.2%) in the olaparib maintenance therapy group in our study was very similar to that of different studies (4, 9, 10, 26). The rates of grade 3–4 hematological AEs, dose reduction (23.5%), dose interruption (23.5%) and treatment discontinuation in patients receiving niraparib were generally lower than those reported in the phase 3 NORA trial (7), and were comparable to those observed in a real-world study in China (27). Lower dose reduction and interruption rates may account for the lower AE rate in our study.

The incidence of leucopenia and thrombocytopenia of any grade was higher for patients receiving niraparib compared with those receiving olaparib but did not differ between the groups of grade 3 or worse severity. Hematological AEs, especially thrombocytopenia, are more frequent with niraparib in comparison with olaparib as reported in previous randomized trials (4, 6). The retrospective RADAR analysis and data from NORA (7) proved that an individualized starting dose (ISD) of niraparib based on the baseline bodyweight and platelet count of patients led to a lower incidence of hematological toxicity without compromising treatment effectiveness. The fact that all patients initiated niraparib maintenance treatment at a fixed starting dose of 200 mg once a day at our hospital may have resulted in a further reduction of hematological toxicity. Our results suggest that olaparib and niraparib are safe and well tolerated by the Chinese population in a real-world setting.

There are several potential limitations to the current study, including its retrospective design and selection biases. The use of single-center study data with poor generalizability could also be considered a limitation of our study. Another key point is that the sample size was not large enough to allow for more specific subgroup analysis, and the follow-up time was not long enough to determine overall survival outcomes. Finally, more than 30% of patients in the cohort did not perform BRCA genetic testing. The absence of BRCA mutation status affects the interpretation of conclusions to some extent.

In conclusion, we found that maintenance treatment with olaparib and niraparib is effective and well tolerated for PSROC patients in real-world clinical practice. Three clinical factors predictive of prolonged survival under PARP inhibitor maintenance therapy, which are easily accessible in routine practice, were identified: BRCA mutant type, PFI ≥12 months, and CR to the last platinum-based therapy. These findings should be further confirmed with an appropriately powered analysis in studies with larger sample sizes.

## Data availability statement

The raw data supporting the conclusions of this article will be made available by the authors, without undue reservation.

## Ethics statement

This study was reviewed and approved by the Ethical Committee of Shandong Cancer Hospital and Institute. Written informed consent for participation was not required for this study in accordance with the national legislation and the institutional requirements.

## Author contributions

Conception and design: SZ and DZ. Development of methodology: DZ. Acquisition of data: DZ, SL, XZ, and JP. Statistical analysis and interpretation of data: DZ and SZ. Writing and revision of the manuscript: DZ and SZ. Funding acquisition: SZ. All authors listed have made a substantial, direct, and intellectual contribution to the work and approved it for publication.

## Funding

This work was supported by the National Natural Science Foundation of China (81972437).

## Conflict of interest

The authors declare that the research was conducted in the absence of any commercial or financial relationships that could be construed as a potential conflict of interest.

## Publisher’s note

All claims expressed in this article are solely those of the authors and do not necessarily represent those of their affiliated organizations, or those of the publisher, the editors and the reviewers. Any product that may be evaluated in this article, or claim that may be made by its manufacturer, is not guaranteed or endorsed by the publisher.
