# Supplementary material for: What predicts the clinical benefits of PARP inhibitors in platinum-sensitive recurrent ovarian cancer: A real-world single-center retrospective cohort study from China
Source: Front Oncol. 2022 Aug 18;12:955124. doi: 10.3389/fonc.2022.955124 (PMC9433773; doi:10.3389/fonc.2022.955124)
Supplement: Supplementary file 1 [file Image_1.pdf]

Figure S1. Three factors associated with prolonged chemotherapy-free interval (CFI) under PARP inhibitors maintenance therapy in patients with platinum-sensitive recurrent ovarian cancer were identified: BRCA mutant type (A), PFI ≥12 months (B) and CR to last platinum-based therapy (C). PARP inhibitor regimen was not an independent prognostic factor for CFI(D).

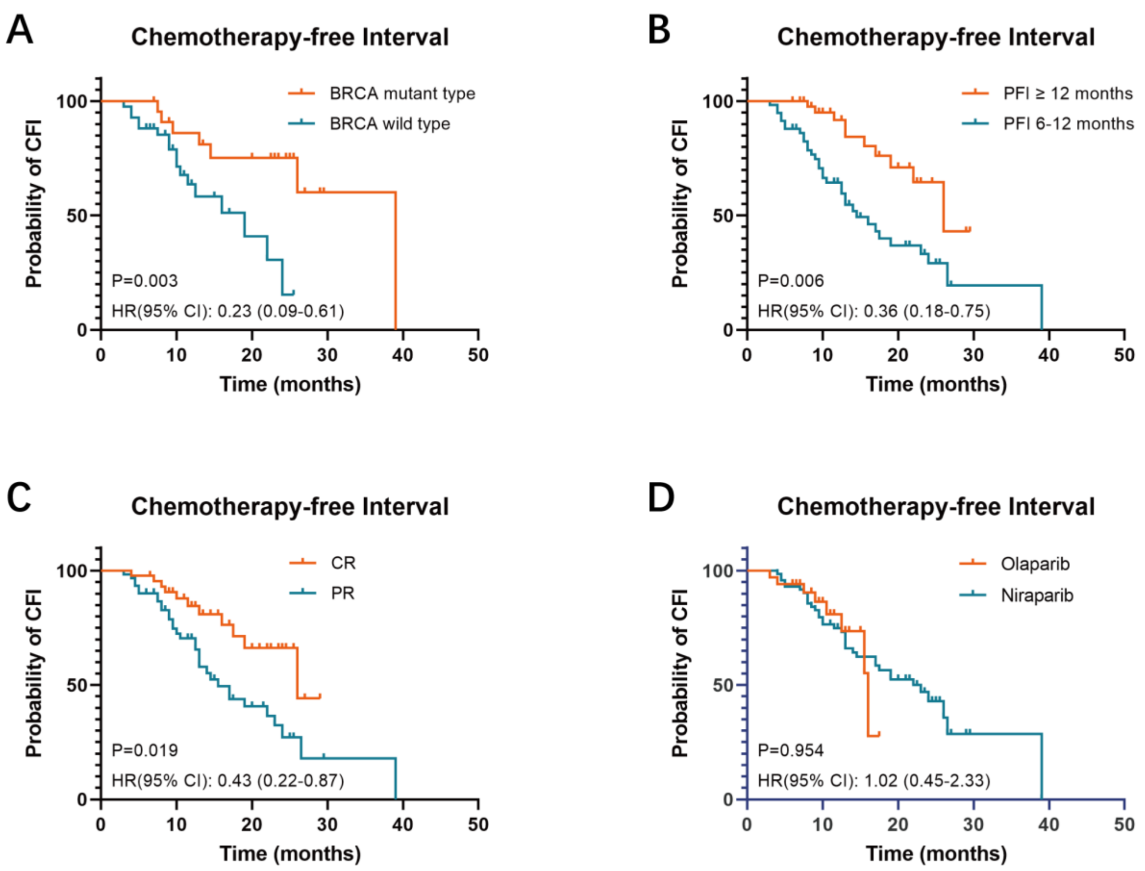

Abbreviations: BRCA, breast cancer susceptibility gene; PARP, poly ADP-ribose polymerase; PFI, platinum-free interval; CR, complete response; PR, partial response; HR, hazard ratios, CI, confidence intervals.
