# Supplementary material for: What predicts the clinical benefits of PARP inhibitors in platinum-sensitive recurrent ovarian cancer: A real-world single-center retrospective cohort study from China
Source: Front Oncol. 2022 Aug 18;12:955124. doi: 10.3389/fonc.2022.955124 (PMC9433773; doi:10.3389/fonc.2022.955124)
Supplement: Supplementary file 3 [file Table_1.docx]

Table S1. Univariate and multivariate analysis of chemotherapy-free interval for the entire cohort.

| **Clinical factors** | **Univariate analysis** | |  | **Multivariate analysis** | |
| --- | --- | --- | --- | --- | --- |
|  | ***HR (95% CI)*** | ***P*** |  | ***HR (95% CI)*** | ***P*** |
| **PARP inhibitors** |  |  |  |  |  |
| Olaparib vs Niraparib | 0.98 (0.45-2.12) | 0.954 |  |  |  |
| **Age** |  |  |  |  |  |
| < 55 vs ≥ 55 | 0.88 (0.48-1.60) | 0.659 |  |  |  |
| **BRCA mutation** |  |  |  |  |  |
| Mutant vs Wild | **0.35 (0.16-0.78)** | **0.008** |  | **0.23 (0.09-0.61)** | **0.003** |
| Unknown vs Wild | 0.73 (0.37-1.42) | 0.329 |  |  |  |
| **Stage** |  |  |  |  |  |
| III-IV vs I-II | 0.67 (0.33-1.37) | 0.214 |  |  |  |
| **Macroscopic residual disease** |  |  |  |  |  |
| Absent vs Present | 0.86 (0.44-1.67) | 0.637 |  |  |  |
| **Number of previous lines of platinum-based therapy** |  |  |  |  |  |
| 2 vs ≥3 | 1.16 (0.62-2.16) | 0.628 |  |  |  |
| **Secondary cytoreductive surgery** |  |  |  |  |  |
| Yes vs No | 0.87 (0.38-1.99) | 0.753 |  |  |  |
| **PFI after the penultimate platinum-based therapy** |  |  |  |  |  |
| ≥12 months vs 6-12 months | **0.33 (0.18-0.60)** | **0.001** |  | **0.36 (0.18-0.75)** | **0.006** |
| **Overall response to last platinum-based therapy** |  |  |  |  |  |
| CR vs PR | **0.41 (0.22-0.75)** | **0.007** |  | **0.43 (0.22-0.87)** | **0.019** |
| **CA-125 response** |  |  |  |  |  |
| Yes vs No | 0.68 (0.36-1.26) | 0.192 |  |  |  |
| **Combined with bevacizumab in last platinum-based therapy** |  |  |  |  |  |
| Yes vs No | 1.22 (0.59-2.50) | 0.568 |  |  |  |

Abbreviations: PARP, Poly ADP-ribose Polymerase; PFI, platinum-free interval.
