# Supplementary material for: What predicts the clinical benefits of PARP inhibitors in platinum-sensitive recurrent ovarian cancer: A real-world single-center retrospective cohort study from China
Source: Front Oncol. 2022 Aug 18;12:955124. doi: 10.3389/fonc.2022.955124 (PMC9433773; doi:10.3389/fonc.2022.955124)
Supplement: Supplementary file 4 [file Table_2.docx]

Table S2: Univariate and multivariate analysis of progression-free survival for the olaparib subgroup.

| **Clinical factors** | **Univariate analysis** | |  | **Multivariate analysis** | |
| --- | --- | --- | --- | --- | --- |
|  | ***HR (95% CI)*** | ***P*** |  | ***HR (95% CI)*** | ***P*** |
| **Age** |  |  |  |  |  |
| < 55 vs ≥ 55 | 0.74 (0.38-1.44) | 0.355 |  |  |  |
| **BRCA mutation** |  |  |  |  |  |
| Mutant vs Wild | **0.33 (0.12-0.87)** | **0.014** |  | **0.31 (0.11-0.84)** | **0.021** |
| **Stage** |  |  |  |  |  |
| III-IV vs I-II | 0.56 (0.25-1.24) | 0.091 |  |  |  |
| **Macroscopic residual disease** |  |  |  |  |  |
| Absent vs Present | 0.75 (0.36-1.57) | 0.398 |  |  |  |
| **Number of previous lines of platinum-based therapy** |  |  |  |  |  |
| 2 vs ≥3 | 1.14 (0.58-2.24) | 0.686 |  |  |  |
| **Secondary cytoreductive surgery** |  |  |  |  |  |
| Yes vs No | 1.24 (0.48-3.19) | 0.625 |  |  |  |
| **PFI after the penultimate platinum-based therapy** |  |  |  |  |  |
| ≥12 months vs 6-12 months | **0.40 (0.20-0.76)** | **0.013** |  | **0.46 (0.20-1.03)** | **0.058** |
| **Overall response to last platinum-based therapy** |  |  |  |  |  |
| CR vs PR | **0.43 (0.22-0.84)** | **0.022** |  | **0.43 (0.20-0.93)** | **0.032** |
| **CA-125 response** |  |  |  |  |  |
| Yes vs No | 0.73 (0.36-1.47) | 0.344 |  |  |  |
| **Combined with bevacizumab in last platinum-based therapy** |  |  |  |  |  |
| Yes vs No | 1.03 (0.45-2.36) | 0.951 |  |  |  |

Abbreviations: BRCA, breast cancer susceptibility gene; PARP, Poly ADP-ribose Polymerase; PFI, platinum-free interval.
